# Impact of COVID‐19 on Venous Thromboembolism in Inflammatory Bowel Disease Hospitalizations: A Propensity‐Matched Analysis

**DOI:** 10.1002/jgh3.70220

**Published:** 2025-07-11

**Authors:** Mihir Prakash Shah, Dushyant Singh Dahiya, Pius Ojemolon, Charmy Parikh, Yash Shah, Bhanu Siva Mohan Pinnam, Manesh Kumar Gangwani, Hassam Ali, Chun‐Wei Pan, Ruchir Paladiya, Abdul Mohammed, Saurabh Chandan, Benjamin Mba, Babu P. Mohan

**Affiliations:** ^1^ Department of Internal Medicine University of Oklahoma Oklahoma City Oklahoma USA; ^2^ Division of Gastroenterology, Hepatology & Motility The University of Kansas School of Medicine Kansas City Kansas USA; ^3^ Department of Gastroenterology and Hepatology Emory University Hospital Georgia USA; ^4^ Department of Internal Medicine Mercy Catholic Medical Center Darby Pennsylvania USA; ^5^ Division of Internal Medicine Trinity Health Oakland/Wayne State University Pontiac Michigan USA; ^6^ Department of Internal Medicine Cook County Health Chicago Illinois USA; ^7^ Department of Gastroenterology and Hepatology University of Arkansas for Medical Sciences Little Rock Arkansas USA; ^8^ Division of Gastroenterology, Hepatology and Nutrition East Carolina University/Brody School of Medicine Greenville North Carolina USA; ^9^ Department of Internal Medicine University of Connecticut Health Center Farmington Connecticut USA; ^10^ Department of Gastroenterology and Hepatology Advent Health Orlando Florida USA; ^11^ Center for Interventional Endoscopy, Advent Health Orlando Florida USA; ^12^ Advanced Endoscopy, Houston Methodist West Hospital Houston Texas USA; ^13^ Department of Internal Medicine Yale School of Medicine New Haven Connecticut USA; ^14^ Department of Gastroenterology Orlando Gastroenterology PA Orlando Florida USA

**Keywords:** COVID‐19, inflammatory bowel disease, NIS, venous thromboembolism

## Abstract

**Background and Aim:**

Patients diagnosed with Inflammatory bowel disease (IBD) face a notably higher risk of venous thromboembolism (VTE), leading to significant health challenges. Similarly, coronavirus disease 2019 (COVID‐19) is associated with an increased susceptibility to thrombosis. We aimed to assess the impact of COVID‐19 on the risk of developing VTE in patients with an underlying diagnosis of IBD.

**Methods:**

We retrospectively analyzed the National Inpatient Sample (NIS) 2020–21 to identify adult patients with IBD admitted with or without a principal diagnosis of COVID‐19. We divided these patients into three groups (without COVID‐19, with uncomplicated COVID‐19, and with complicated COVID‐19). Hospitalization characteristics, in‐hospital mortality, odds of VTE, healthcare burden, and complications were compared.

**Results:**

IBD patients with complicated COVID‐19 infection had significantly higher odds of VTE (OR 5.60, 95% C.I. 3.63–8.65, *p* 0.001), an increase in odds of mortality (OR 29.13, 95% C.I. 22.59–37.57, *p* 0.001), higher healthcare resource utilization (including length of stay and total hospitalization charges), and worse secondary outcomes (like acute kidney injury and pancytopenia), compared to IBD patients without COVID‐19. IBD patients with uncomplicated COVID‐19 also had higher odds of VTE (OR 1.81, 95% C.I. 1.39–2.36, *p* 0.001) than those without COVID‐19; however, there was no difference in mortality or length of stay between these two groups, and those with uncomplicated COVID‐19 had lower average total hospitalization charges.

**Conclusion:**

Patients with both complicated and uncomplicated COVID‐19 were associated with higher odds of VTE compared to those without COVID‐19. Patients with complicated COVID‐19, in addition, also had higher odds of mortality.

## 1 Introduction

The coronavirus disease 2019 (COVID‐19) infection is associated with significant morbidity and mortality [1, 2]. The significant healthcare burden imposed by the COVID‐19 infection is partly due to an increased propensity to thrombosis [3, 4, 5, 6, 7]. Inflammatory bowel disease (IBD) has a high global healthcare burden, with a prevalence of about 6.8 million patients worldwide in 2017 [8]. The United States is home to more than 1 million of those [9], with the highest age‐standardized prevalence (465 per 100 000 population) [8]. The pathogenesis of IBD involves inflammation of the gastrointestinal (GI) tract. It is characterized by a multifaceted interaction of various environmental, genetic, and personal factors, with immune system dysregulation being a key contributing element [10, 11]. At baseline, compared to populations without the disease, patients with IBD have a higher risk of venous thromboembolism (VTE) [12], and the risk is significantly increased during times of IBD flares [13]. The development of VTE in individuals with IBD is attributed to a complex interaction of multiple inherited and acquired risk factors [14].

Given the increased risk of thromboembolic events with both COVID‐19 and IBD, we aimed to assess the impact of COVID‐19 on the risk of developing VTE in patients with an underlying diagnosis of IBD.

## 2 Methods

### 2.1 Data Source

We utilized the National Inpatient Sample (NIS) database to identify patients admitted to acute care hospitals in the US during 2020 and 2021. The NIS is the largest publicly available all‐payer inpatient database developed for the Healthcare Cost and Utilization Project (HCUP) [15]. Hospitals are stratified using the American Hospital Association annual hospital survey. Patient‐level data (including age, sex, race, income in patient's ZIP code, insurance status, principal and secondary diagnoses, procedures performed) and hospital‐level data (teaching status, bed size, urban/rural location, geographic region) can be obtained. The 2020 and 2021 NIS sampling frame includes data from 48 statewide organizations (including the District of Columbia) covering more than 97% of the United States population. Unweighted, it contains data from around 7 million hospital stays each year. When weighted, it estimates around 35 million hospitalizations nationally. It is designated to produce US regional and national estimates of inpatient utilization, access, cost, quality, and outcomes.

### 2.2 Subjects

This is a retrospective cohort study of patients with IBD admitted to the hospital with or without a principal diagnosis of COVID‐19 infection, to assess the impact of COVID‐19 on the odds of VTE. Adult patients with an underlying diagnosis of IBD were included in the study. These patients were divided into groups based on whether the principal discharge diagnosis (defined as the condition chiefly responsible for the patient's admission to the hospital) was COVID‐19 infection. Patients with COVID‐19 infection as the principal discharge diagnosis were subdivided into two groups: those with uncomplicated COVID‐19 and those with complicated COVID‐19. Complicated infections were defined as those associated with sepsis, septic shock, acute respiratory distress syndrome (ARDS), need for non‐invasive ventilatory support (such as positive pressure ventilation, high flow nasal cannula), intubation and mechanical ventilation, or vasopressors. We limited the patient selection to those with the primary discharge diagnosis (DX‐1) of COVID‐19. To further enhance the accuracy of results, patients currently on anticoagulation and those with a history of possible anticoagulation use were excluded. These included patients with a history of VTE, atrial fibrillation or flutter, prosthetic heart valve, primary thrombophilia, intracardiac thrombus, or those with a history of coronary artery bypass graft (CABG) or percutaneous coronary intervention (PCI). Appropriate ICD‐10‐CM (International Classification of Diseases, Tenth Revision, Clinical Modification) codes were used. The ICD‐10‐CM and ICD‐10‐PCS (Procedural Coding System) codes used for the study are provided in the Supporting Information.

### 2.3 Outcomes

Clinical outcomes were compared between 3 groups: (1) IBD patients with uncomplicated COVID‐19 vs. IBD patients without COVID‐19, (2) IBD patients with complicated COVID‐19 vs. IBD patients without COVID‐19, and (3) IBD patients with complicated COVID‐19 vs. IBD patients with uncomplicated COVID‐19. The primary study outcome was in‐hospital all‐cause mortality. Secondary outcomes assessed were: (i) length of stay, (ii) total hospitalization charges, (iii) pulmonary embolism (PE), (iv) deep vein thrombosis (DVT), (v) portal vein thrombosis, (vi) Budd‐Chiari syndrome, (vii) combined VTE, (viii) acute kidney injury (AKI), (ix) acute myocardial infarction (MI), (x) stroke, (xi) GI bleeding, (xii) need for blood transfusion, (xiii) GI perforation, (xiv) acute limb ischemia, (xv) arterial thromboembolism (ATE), (xvi) splenic infarction, (xvii) spinal infarction, (xviii) retinal vessel occlusion, (xix) thrombocytopenia, (xx) pancytopenia, (xxi) thrombocytosis, (xxii) nephrotic syndrome, (xxiii) hypoalbuminemia, (xxiv) skin manifestations of IBD such as erythema nodosum (EN), pyoderma gangrenosum (PG), and hidradenitis suppurativa (HS).

### 2.4 Statistical Analysis

We used STATA (StataCorp LLC, College Station, TX) version 18‐MP for statistical analysis. NIS is based on a complex sampling design, including stratification, clustering, and weighting. STATA facilitates analysis of this patient data to produce nationally representative, unbiased results. In our analysis, we used two methods to adjust for confounders: multivariate regression analysis and propensity score matching. Initially, proportions were compared using Fisher's exact test. An independent sample t‐test was used to compare the mean of continuous data. Univariate regression analysis was performed to obtain an unadjusted odds ratio (OR) for each outcome. Based on the univariate screen significance (*p* < 0.2), we selected the variables to perform the multivariate logistic regression analysis to adjust for confounders. The final multivariate model was adjusted for the following variables: (1) demographic variables: age, gender, insurance status, hospital bed size, teaching status, hospital location (urban/rural), zip‐code‐wise median income status; (2) comorbid variables: Elixhauser comorbidity index, anemia, diabetes mellitus, hypertension, smoking, obesity, chronic/end‐stage kidney disease, congestive heart failure, dyslipidemia, malnutrition, Chronic Obstructive Pulmonary Disease, cancer, chronic lung diseases, cirrhosis, carotid atherosclerotic disease, peripheral artery disease, and coronary artery disease. Logistic regression was used for binary outcomes and linear regression was used for continuous outcomes. In addition, propensity scores were used to match patients in all three groups one to one. A non‐parsimonious multivariate regression model was developed to estimate the propensity score using multiple demographic and comorbidity covariates. The double robust method was then used to generate treatment weights, followed by inverse probability of treatment weighting to match cases to controls using generalized linear models. The propensity‐matched cohort of patients was then used to obtain clinical outcomes. In particular, cancer was balanced between the comparison groups in all three sub‐group analyses as it had a statistically significant difference in prevalence (10%, 40%, and 60%), and it is a strong and well‐recognized risk factor of VTE. All *p*‐values were two‐sided, with 0.05 as the threshold for significance. Multiple imputation was not required for the analysis, as there were a negligible number of missing values for all the potential confounders (the highest being 1.5%).

## 3 Ethical Considerations

The data in NIS is de‐identified to protect patient privacy. Since 2012, the NIS has also removed state‐level and hospital identifiers. Hence, our study was exempt from Institutional Review Board (IRB) approval.

## 4 Results

### 4.1 Patient and Hospital Characteristics

**FIGURE 1:**
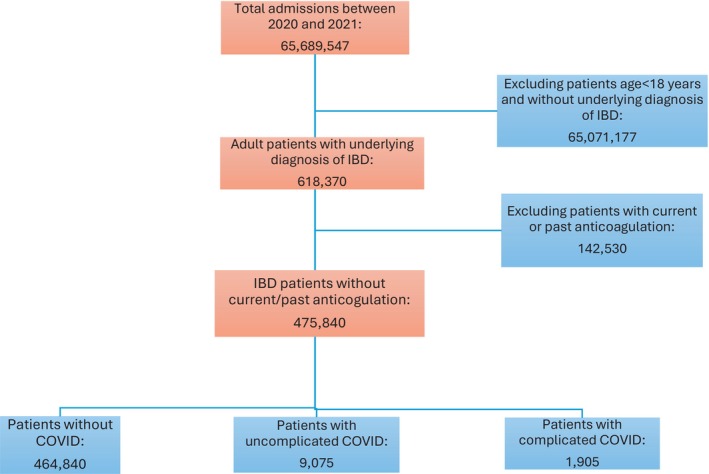
Adult patients with inflammatory bowel disease without current/past anticoagulation use between 2020 and 2021, divided into groups based on principal discharge diagnosis of COVID‐19.

Between 2020 and 2021, there were approximately 65.69 million hospital admissions in the United States. Out of this, 475 840 were adult patients with an underlying diagnosis of IBD without a current or past therapy with anticoagulation (see Figure 1). From this study population, 9075 (1.9%) patients had a principal discharge diagnosis of uncomplicated COVID‐19, and another 1905 (0.4%) patients had complicated COVID‐19 as the principal discharge diagnosis.

**Table:** 

Characteristic	No COVID‐19	Uncomplicated COVID‐19	Complicated COVID‐19	*p*
Mean age	51.64	60.13	63.78	0.001
Age category (years)				0.001
18–45	39.20%	17.30%	8.90%	
45–65	32.00%	41.30%	37.30%	
> 65	28.80%	41.40%	53.80%	
Gender				0.012
Male	43.00%	44.80%	49.60%	
Female	57.00%	55.20%	50.40%	
Race				0.877
White	74.70%	75.40%	76.60%	
Black	11.80%	11.50%	9.70%	
Hispanics	7.00%	6.90%	7.60%	
Others	6.50%	6.20%	6.00%	
Insurance status				0.001
Medicare	38.30%	48.90%	59.80%	
Medicaid	17.40%	10.40%	6.80%	
Private insurance	40.30%	38.60%	32.60%	
Self‐pay	4.00%	2.10%	0.80%	
Elixhauser comorbidity index				0.001
0	12.80%	5%	1%	
1	18.10%	15.40%	4.70%	
2	18.70%	20.20%	10.20%	
3 or more	50.40%	59.00%	83.70%	
Median household income in patient's ZIP code (quartile)				0.001
1st (0–25)	24.90%	26.70%	28.90%	
2nd (26–50)	25.70%	27.30%	26.00%	
3rd (51–75)	25.00%	26.30%	27.60%	
4th (76–100)	24.40%	19.70%	17.50%	
Hospital bed size				0.001
Small	22.10%	28.00%	24.70%	
Medium	26.80%	27.30%	33.60%	
Large	51.10%	44.70%	41.70%	
Hospital teaching status				0.001
Non‐teaching	23.20%	31.10%	31.20%	
Teaching	76.80%	68.90%	68.80%	
Hospital location				0.001
Rural	6.90%	11.90%	12.90%	
Urban	93.10%	88.10%	87.10%	
Anemia	36.10%	20.10%	33.60%	0.001
DM	16.80%	27.50%	33.60%	0.001
HTN	41.20%	55.90%	68.00%	0.001
Smoking	38.60%	36.70%	32.80%	0.016
Obesity	14.40%	24.60%	37.00%	0.001
CKD/ESRD	11.90%	14.40%	17.30%	0.001
CHF	8.00%	8.10%	18.90%	0.001
Dyslipidemia	23.30%	36.40%	38.60%	0.001
Malnutrition	13.40%	5.90%	13.90%	0.001
COPD	10.60%	15.00%	22.60%	0.001
Cancer	10.60%	40.20%	60.40%	0.001
Chronic lung disease	19.50%	27.20%	32.60%	0.001
Cirrhosis	3.30%	2.30%	3.20%	0.065
Steroid use	6.00%	5.50%	6.60%	0.605
Contraceptive use	0.03%	0.00%	0.00%	0.745
Vasculitides	2.40%	2.20%	2.60%	0.985
Carotid atherosclerotic disease	0.60%	0.20%	1.10%	0.03
Renal atherosclerotic disease	0.10%	0.10%	0.00%	0.812
HIV	0.70%	0.50%	0.30%	0.436
Peripheral artery disease	2.60%	2.50%	4.20%	0.124
Coronary artery disease	10.10%	15.60%	16.80%	0.001

Patients with COVID‐19 were likely to be much older than those without COVID‐19 (60.1 years for uncomplicated COVID‐19 and 63.8 years for complicated COVID‐19, vs. 51.6 years for patients without COVID‐19; *p* 0.001). They were also less likely to be female (55% and 50%, vs. 57%; *p* 0.012), more likely to be on Medicare insurance (49% and 60%, vs. 38%; *p* 0.01), and more likely to be treated at smaller, non‐teaching, rural hospitals compared to patients without COVID‐19 [see Table 1]. Racial profiles between the three groups were similar.

**TABLE 1:** Baseline characteristics between the three groups.

Outcome	IBD with uncomplicated COVID‐19 vs. IBD without COVID‐19		IBD with complicated COVID‐19 vs. IBD without COVID‐19		IBD with complicated COVID‐19 vs. IBD with UNCOMPLICATED COVID‐19	
	odds ratio (95% C.I.) [unless otherwise specified]	*p*‐value	odds ratio (95% C.I.) [unless otherwise specified]	*p*‐value	odds ratio (95% C.I.) [unless otherwise specified]	*p*‐value
Mortality						
Adjusted	1.13 (0.78 to 1.64)	0.528	**38 (29.36 to 49.18)**	**0.001**	**37.83 (23.87 to 59.98)**	**0.001**
Matched	0.75 (0.51 to 1.13)	0.17	**29.13 (22.59 to 37.57)**	**0.001**	**33.1 (21.54 to 50.86)**	**0.001**
Length of stay [days]						
Adjusted	**0.93 (0.69 to 1.17)**	**0.001**	**9.64 (8.40 to 10.87)**	**0.001**	**8.5 (7.30 to 9.69)**	**0.001**
Matched	0.07 (−0.21 to 0.35)	0.628	**9.63 (7.34 to 11.91)**	**0.001**	**9.07 (7.57 to 10.57)**	**0.001**
Total hospitalization charges [USD]						
Adjusted	**−6915 (−9614 to −4217)**	**0.001**	**128 280 (103 719 to 152 841)**	**0.001**	**135 088 (112 836 to 157 342)**	**0.001**
Matched	**−16 658 (−19 789 to −13 528)**	**0.001**	**116 584 (79 713 to 153 455)**	**0.001**	**115 074 (96 862 to 133 287)**	**0.001**
Pulmonary embolism						
Adjusted	**3 (2.26 to 4.00)**	**0.001**	**4.86 (3.23 to 7.32)**	**0.001**	**2.1 (1.26 to 3.49)**	**0.004**
Matched	**3.12 (2.27 to 4.31)**	**0.001**	**5.79 (3.45 to 9.74)**	**0.001**	**2 (1.21 to 3.31)**	**0.007**
Deep vein thrombosis						
Adjusted	1.4 (0.98 to 2.00)	0.065	**4.17 (2.74 to 6.33)**	**0.001**	**4.08 (2.27 to 7.31)**	**0.001**
Matched	1.03 (0.70 to 1.52)	0.889	**5.75 (3.30 to 10.04)**	**0.001**	**4.09 (2.36 to 7.11)**	**0.001**
Combined VTE						
Adjusted	**2.15 (1.70 to 2.72)**	**0.001**	**4.8 (3.47 to 6.64)**	**0.001**	**2.89 (1.92 to 4.36)**	**0.001**
Matched	**1.81 (1.39 to 2.36)**	**0.001**	**5.6 (3.63 to 8.65)**	**0.001**	**2.79 (1.87 to 4.16)**	**0.001**
Acute kidney injury						
Adjusted	1.01 (0.89 to 1.16)	0.849	**2.85 (2.27 to 3.58)**	**0.001**	**2.92 (2.24 to 3.82)**	**0.001**
Matched	0.95 (0.82 to 1.10)	0.505	**2.83 (2.06 to 3.89)**	**0.001**	**2.87 (2.16 to 3.79)**	**0.001**
Acute myocardial infarction						
Adjusted	0.65 (0.42 to 1.00)	0.05	1.52 (0.89 to 2.62)	0.127	**2.74 (1.44 to 5.18)**	**0.002**
Matched	**0.6 (0.37 to 0.96)**	**0.035**	1.44 (0.88 to 2.36)	0.144	**2.5 (1.29 to 4.85)**	**0.007**
Stroke						
Adjusted	**0.17 (0.06 to 0.46)**	**0.001**	0.8 (0.33 to 1.96)	0.626	9.97 (0.48 to 208.50)	0.138
Matched	**0.19 (0.06 to 0.58)**	**0.003**	0.9 (0.37 to 2.16)	0.81	3.62 (0.87 to 15.07)	0.077
GI bleeding						
Adjusted	**0.41 (0.32 to 0.51)**	**0.001**	0.71 (0.49 to 1.03)	0.073	**1.63 (1.02 to 2.62)**	**0.043**
Matched	**0.32 (0.25 to 0.41)**	**0.001**	**0.41 (0.27 to 0.63)**	**0.001**	1.3 (0.81 to 2.07)	0.273
Blood transfusions						
Adjusted	**0.29 (0.18 to 0.047)**	**0.001**	**1.7 (1.12 to 2.59)**	**0.013**	**6.44 (3.25 to 12.77)**	**0.001**
Matched	**0.19 (0.11 to 0.32)**	**0.001**	1.97 (0.96 to 4.07)	0.066	**6.92 (3.46 to 13.84)**	**0.001**
Arterial thromboembolism						
Adjusted	N/A		3.07 (0.78 to 12.02)	0.107	N/A	
Matched	N/A		1.58 (0.37 to 6.69)	0.535	N/A	
Hypoalbuminemia						
Adjusted	1.22 (0.80 to 1.85)	0.352	**2.07 (1.13 to 3.80)**	**0.018**	1.71 (0.83 to 3.56)	0.147
Matched	0.84 (0.55 to 1.28)	0.412	1.1 (0.54 to 2.22)	0.798	1.56 (0.78 to 3.14)	0.21
Thrombocytopenia						
Adjusted	**2.2 (1.78 to 2.70)**	**0.001**	**2.54 (1.82 to 3.54)**	**0.001**	1.3 (0.88 to 1.94)	0.19
Matched	**1.74 (1.39 to 2.19)**	**0.001**	**1.81 (1.24 to 2.64)**	**0.002**	1.12 (0.76 to 1.64)	0.578
Pancytopenia						
Adjusted	**2.87 (1.98 to 4.17)**	**0.001**	**3.24 (1.80 to 5.84)**	**0.001**	1.31 (0.63 to 2.74)	0.475
Matched	1.4 (0.97 to 2.03)	0.072	**7.73 (2.60 to 23.05)**	**0.001**	1.46 (0.72 to 2.95)	0.298
Thrombocytosis						
Adjusted	1.24 (0.46 to 3.30)	0.669	1.26 (0.18 to 8.90)	0.82	0.33 (0.01 to 12.28)	0.548
Matched	0.61 (0.21 to 1.78)	0.366	1.03 (0.17 to 6.03)	0.969	1.08 (0.13 to 8.99)	0.941

Patients with complicated COVID‐19 (84%) were much more likely to have three or more comorbidities than those with uncomplicated COVID‐19 (59%) and those without COVID‐19 (50%), with a *p*‐value of 0.001. They were also more likely to have diabetes mellitus (33.6% vs. 27.5% and 16.8% respectively; *p* 0.001), hypertension (68% vs. 55.9% and 41.2% respectively; *p* 0.001), obesity (37% vs. 24.6% and 14.4% respectively; *p* 0.001), advanced kidney disease (17.3% vs. 14.4% and 11.9% respectively; *p* 0.001), chronic lung diseases (32.6% vs. 27.2% and 19.5% respectively; *p* 0.001), heart failure (18.9% vs. 8.1% and 8% respectively; *p* 0.001), and carotid atherosclerotic disease burden (1.1% vs. 0.2% and 0.6% respectively; *p* 0.001), compared to the other two groups. In addition, they were much more likely to have cancer (60% compared to 40% and 10% respectively, *p* 0.001), which is a notable baseline difference as cancer by itself is also known to increase the likelihood of developing VTE.

### 4.2 Outcomes

Primary analysis was performed using multivariate regression to adjust for possible confounders identified by the univariate regression screening. An important consideration was the significant difference in the total number of patients with COVID‐19 (only 2.3%) compared to those without COVID‐19 (97.7%) in the IBD population. To account for this difference, additional analysis using propensity score matching was performed to match these patients individually to further enhance our results' accuracy.

#### 4.2.1 Primary Outcome (Mortality)

Patients with complicated COVID‐19 infection had a manifold increase in odds of mortality compared to those with uncomplicated COVID‐19 infection (OR 33.10, 95% C.I. 21.54–50.86, *p* 0.001) and those without COVID‐19 infection (OR 29.13, 95% C.I. 22.59–37.57, *p* 0.001) [see Table 2]. There was no difference in mortality between patients with uncomplicated COVID‐19 and patients without COVID‐19 (OR 0.75, 95% C.I. 0.51–1.13, *p* 0.17).

**TABLE 2:** Results of sub‐group analysis.

Outcome	Uncomplicated COVID‐19 vs. control (no IBD)		Complicated COVID‐19 vs. control (no IBD)		IBD vs. control‐2 (no COVID‐19)	
	odds ratio (95% C.I.) [unless otherwise specified]	*p*‐value	odds ratio (95% C.I.) [unless otherwise specified]	*p*‐value	odds ratio (95% C.I.) [unless otherwise specified]	*p*‐value
Combined VTE	1.38 (1.36 to 1.41)	0.001	3.01 (2.93 to 3.10)	0.001	1.08 (1.05 to 1.11)	0.001
Pulmonary embolism (PE)	2.04 (2.00 to 2.09)	0.001	2.92 (2.83 to 3.02)	0.001	0.89 (0.85 to 0.94)	0.001
Deep vein thrombosis (DVT)	0.78 (0.76 to 0.81)	0.001	2.66 (2.56 to 2.76)	0.001	1.15 (1.11 to 1.19)	0.001

#### 4.2.2 Secondary Outcomes

Patients with complicated COVID‐19 were five times more likely (OR 5.60, 95% C.I. 3.63–8.65, *p* 0.001) to have VTE compared to patients without COVID‐19, and almost three times more likely (OR 2.79, 95% C.I. 1.87–4.16, *p* 0.001) compared to patients with uncomplicated COVID‐19. This was due to increased odds of having both PE and DVT. For patients with complicated COVID‐19, the odds of having PE were higher compared to both patients without COVID‐19 (OR 5.79, 95% C.I. 3,45–9.74, *p* 0.001) and patients with uncomplicated COVID‐19 (OR 2.00, 95% C.I. 1.21–3.31, *p* 0.007). These patients also had higher odds of developing DVT compared to patients without COVID‐19 (OR 5.75, 95% C.I. 3.30–10.04, *p* 0.001) and those with uncomplicated COVID‐19 infection (OR 4.09, 95% C.I. 2.36–7.11, *p* 0.001). In comparison to patients without COVID‐19, patients with uncomplicated COVID‐19 were almost twice as likely to have VTE (OR 1.81, 95% C.I. 1.39–2.36, *p* 0.001), and this seemed to be driven primarily by increased odds of PE (OR 3.12, 95% C.I. 2.27–4.31, *p* 0.001), with no statistically significant difference in the odds of DVT (OR 1.03, 95% C.I. 0.70–1.52, *p* 0.889).

Compared to patients without COVID‐19, those with complicated COVID‐19 had increased odds of AKI (OR 2.83, 95% C.I. 2.06–3.89, *p* 0.001), thrombocytopenia (OR 1.81, 95% C.I. 1.24–2.64, *p* 0.002), and pancytopenia (OR 7.73, 95% C.I. 2.60–23.05, *p* 0.001) while having lower odds of GI bleeding (OR 0.41, 95% C.I. 0.27–0.63, *p* 0.001). The odds of acute MI (OR 1.44, 95% C.I. 0.88–2.36, *p* 0.144), stroke (OR 0.90, 95% C.I. 0.37–2.16, *p* 0.81), ATE (OR 1.58, 95% C.I. 0.37–6.69, *p* 0.535), and thrombocytosis (OR 1.03, 95% C.I. 0.17–6.03, *p* 0.969) were similar.

In contrast to patients with uncomplicated COVID‐19, patients with complicated COVID‐19 were more likely to have AKI (OR 2.87, 95% C.I. 2.16–3.79, *p* 0.001), acute MI (OR 2.50, 95% C.I. 1.29–4.85, *p* 0.007), and require blood transfusions (OR 6.92, 95% C.I. 3.46–13.84, *p* 0.001). However, there was no difference in the odds of developing stroke (OR 3.62, 95% C.I. 0.87–15.07, *p* 0.077), GI bleeding (OR 1.3, 95% C.I. 0.81–2.07, *p* 0.273), pancytopenia (OR 1.46, 95% C.I. 0.72–2.95, *p* 0.298), thrombocytopenia (OR 1.12, 95% C.I. 0.76–1.64, *p* 0.578) or thrombocytosis (OR 1.08, 95% C.I. 0.13–8.99, *p* 0.941).

Patients with uncomplicated COVID‐19 had higher odds of VTE (OR 1.81, 95% C.I. 1.39–2.36, *p* 0.001) along with higher odds of thrombocytopenia (OR 1.74, 95% C.I. 1.39–2.19, *p* 0.001) compared to those without COVID‐19, while having lower odds of acute MI (OR 0.60, 95% C.I. 0.37–0.96, *p* 0.035), stroke (OR 0.19, 95% C.I. 0.06–0.58, *p* 0.003), GI bleeding (OR 0.32, 95% C.I. 0.25–0.41, *p* 0.001), and the need for blood transfusions (OR 0.19, 95% C.I. 0.11–0.32, *p* 0.001). Overall odds of mortality between the two groups were similar (OR 0.75, 95% C.I. 0.51–1.13, *p* 0.17).

### 4.3 Healthcare Burden

Patients with complicated COVID‐19 had significantly higher healthcare resource utilization, with longer average lengths of stay [9.63 days (95% C.I. 7.34–11.91, *p* 0.001) and 9.07 days (95% C.I. 7.57–10.57, *p* 0.001) longer compared to those without COVID‐19 and those with uncomplicated COVID‐19, respectively] and higher average total hospitalization charges [116 584 USD (95% C.I. 79 713–153 455, *p* 0.001) and 115 074 USD (95% C.I. 96 862‐133 287, *p* 0.001) more, respectively]. Combined with the much greater odds of mortality, this reinforces the public healthcare crisis that the COVID‐19 pandemic has led to.

Surprisingly, patients with uncomplicated COVID‐19 had lower average total hospitalization charges (−16 658 USD, 95% C.I. −19 789 to 13 528, *p* 0.001) and similar average length of stay (0.07 days, 95% C.I. −0.21 to 0.35, *p* 0.628) compared to patients without COVID‐19.

### 4.4 Secondary Analysis

Secondary analysis (Table 2) aimed at identifying the independent odds of VTE due to IBD and COVID‐19 was also done. IBD [OR 1.08 (95% C.I. 1.05 to 1.11, *p* = 0.001)], uncomplicated COVID‐19 [OR 1.38 (95% C.I. 1.36 to 1.41, *p* = 0.001)], as well as complicated COVID‐19 [OR 3.01 (95% C.I. 2.93 to 3.10, *p* = 0.001)] all had higher odds of VTE compared to patients without these conditions. For patients with COVID‐19, this was driven primarily by increased odds of PE, while for those with IBD, it was driven by increased odds of DVT. Notably, the odds of VTE in patients who had both IBD and complicated COVID‐19 were higher than the odds due to IBD alone or complicated COVID‐19 alone. This was also true in the case of patients with IBD and uncomplicated COVID‐19.

## 5 Discussion

A complex yet intricate relationship exists between COVID‐19 and IBD as they share specific pathophysiologic mechanisms, including dysregulated innate/adaptive immune responses and frequent need for treatment with immunosuppressive medications [16]. Up to 35% of patients with COVID‐19 infection manifest GI symptoms that are indistinguishable from a clinical presentation of acute IBD. There is conflicting evidence on the impact of IBD in patients with COVID‐19 infection. A prospective cohort study conducted in Italy showed that active IBD was associated with worse outcomes among patients with COVID‐19 [17]. In contrast, a case series of over 1900 patients showed no difference in COVID‐19‐associated mortality in IBD patients [18]. A meta‐analysis of 86 studies showed that IBD was not significantly associated with increased risk of COVID‐19 infection, COVID‐19 hospitalization, severe COVID‐19, or mortality [19].

In our study, the proportion of IBD‐related hospitalizations in 2020–21 was 0.95%. Of these, after excluding patients with current or past anticoagulation use, 2.3% had COVID‐19 infection. Older age and higher comorbidity index were more commonly seen among IBD patients with COVID‐19 infection and even more so for those with complicated COVID‐19 infection. This is similar to prior reports that have shown that age and multiple medical comorbidities are associated with more complications and worse outcomes in cases of COVID‐19 [17]. We found that patients with IBD and complicated COVID‐19 infection had about 29 times higher odds of mortality compared to those without COVID‐19 infection. Still, there was no difference in mortality between patients with uncomplicated COVID‐19 infection and patients without COVID‐19. This significantly higher mortality in patients with complicated COVID‐19 is likely due to complications of COVID‐19 itself and unlikely to be due to IBD‐related factors, as prior studies have demonstrated that IBD does not impact the severity or outcomes of hospitalization with COVID‐19 infection [19]. Compared to patients without COVID‐19, patients with IBD admitted with uncomplicated COVID‐19 had no difference in length of stay. On the other hand, patients with complicated COVID‐19 had significantly longer lengths of hospital stay (over 9 more days on average) compared to those without COVID‐19, as well as higher total hospitalization charges (over $116 000 on average).

Extensive cohort studies and meta‐analyses have revealed that individuals with IBD face a 2–3 times increased risk of experiencing DVT or PE in contrast to those without IBD, and this risk is even higher in those with active disease, flare‐ups needing steroid treatment, those who undergo surgery, and during hospitalization [12, 13, 20, 21]. The exact mechanism underlying the heightened VTE risk in individuals with IBD remains to be fully elucidated. This risk is believed to be multifaceted, influenced by genetics and environment, prothrombotic states, and endothelial dysfunction. The chronic inflammation in IBD leads to increased production of pro‐inflammatory cytokines, including tumor necrosis factor‐alpha (TNF‐α), interleukin‐1 (IL‐1), and IL‐6, which in turn triggers heightened expression of tissue factor and the coagulation cascade. The inflammation also amplifies platelet activation and aggregation, contributing to a hypercoagulable state. The increased likelihood of VTE in IBD patients may also be related to surgery, medications such as corticosteroids and Janus kinase inhibitors, and the role of bacterial components and gut microbiota [22, 23]. The prevalence and incidence rate of all VTE in patients with IBD was 5.6% and 6.3 per 1000 person‐years, respectively. Over 75% of these cases of VTE were unprovoked, and 60.9% occurred in patients with active disease [24]. The probability of recurrence of VTE 5 years after discontinuation of anticoagulation therapy has also been shown to be 2.5 times higher among patients with IBD than in patients without IBD [25]. Patients with COVID‐19 infection are also known to be at high risk of thromboembolism [3, 26, 27, 28]. This risk is considerably higher when hospitalized with severe COVID‐19 infection despite prophylactic anticoagulation [3].

In our study, patients with complicated COVID‐19 were over five times more likely to have PE and DVT compared to patients without COVID‐19, four times more likely to have DVT compared to patients with uncomplicated COVID‐19, and twice as likely as those with uncomplicated COVID‐19 to have PE. In comparison to patients without COVID‐19, patients with uncomplicated COVID‐19 were over three times more likely to have PE but had no statistically significant difference in the odds of DVT. However, there was no difference in the odds of having ATE between patients with complicated COVID‐19 infection and those without COVID‐19. While the reasons behind this interesting finding are unclear and warrant more studies, the risk of ATE is known to be significantly lower than that of VTE in IBD patients, even in the presence of a modestly increased risk of cardiovascular diseases [22, 28, 29]. A meta‐analysis of observational studies showed no increase in the risk of ATE or cardiovascular mortality in IBD patients despite an increased risk of both ischemic heart disease and mesenteric ischemia [20]. It remains unclear how much the higher odds of VTE among patients with IBD and complicated COVID‐19 infection contributed to worse outcomes in this cohort of patients in our study. Prior research conducted with the NIS showed that IBD discharges had higher VTE rates than non‐IBD discharges. VTE was associated with 2.5 times greater mortality among IBD patients, and the age‐ and comorbidity‐adjusted excess mortality from VTE was 2.1‐fold higher for IBD than for non‐IBD patients (*p* < 0.0001). IBD patients with VTE had a longer length of stay (11.7 vs. 6.1 days, *p* < 0.0001) and incurred higher hospital charges ($47 515 vs. $21 499; *p* < 0.0001) in that study [30]. In previous studies, reactive thrombocytosis [31] and hypoalbuminemia [32, 33] have both been associated with an increased risk of VTE. We assessed these secondary outcomes as potential confounders in our results. However, after propensity matching, there was no significant difference in the odds of those outcomes between all three groups in our study.

As mentioned in Table 2, we also performed a secondary analysis to find out the independent odds of VTE due to IBD and COVID‐19 (both complicated and uncomplicated). The results suggested that, although both IBD and COVID‐19 are conditions that increase the odds of VTE in hospitalized patients, for those patients who have IBD at baseline and are hospitalized with COVID‐19, the odds of VTE are significantly higher. This suggests a complex pathophysiological interplay that needs to be investigated further.

Our study has many strengths and some limitations. A key strength of our study is the large, multi‐racial study population, which accurately represents the US population. Hence, our results apply to all hospitalizations across the US. The NIS stores information on up to 25 procedures and 40 diagnosis codes per admission, minimizing the risk of under‐representation of comorbidities. Furthermore, due to our unique multi‐faceted analysis, we provide clinicians with real‐world data on the impact of COVID‐19 on VTE in IBD hospitalizations to identify patients at high risk and aid in clinical decision‐making. However, we acknowledge all the limitations associated with our study. The NIS reports data on hospitalizations, not individual patients; thus, recurrent hospitalizations count as multiple admissions, affecting our reported proportions.

Additionally, vital signs, laboratory, and radiologic data are unavailable; hence, we could not classify COVID‐19 hospitalizations based on severity. However, we made up for this by applying secondary measures that were known complications of the infection. The NIS is an administrative database that relies on the correct input of diagnostic and therapeutic ICD codes, which may not have wholly matched clinical parameters (diagnosis code misclassification). Despite the limitations highlighted, this was a comprehensive study using a large nationally representative cohort, allowing us to provide insights into the outcomes of hospitalizations for COVID‐19 in patients with IBD.

## 6 Conclusion

Among patients with IBD, hospitalizations for complicated COVID‐19 infection were associated with significantly increased odds of developing VTE, increased mortality, as well as increased morbidity and healthcare resource utilization, compared to hospitalizations for patients with uncomplicated COVID‐19 infection or those without COVID‐19 infection. Patients with uncomplicated COVID‐19 infection were also associated with increased odds of developing VTE compared to patients without COVID‐19 infection. However, they did better with multiple other secondary outcomes, ultimately leading to similar odds of mortality between the two groups. Our findings reinforce the significant public healthcare burden the COVID‐19 pandemic has been and highlight that patients with IBD are a very susceptible group with the potential for worse hospitalization outcomes. For patients with IBD who have concomitant COVID‐19 infection, physicians, including gastroenterologists, need to be mindful about disruptions in VTE prophylaxis measures which are often prevalent in the peri‐procedural period (e.g., Patients with IBD flare undergoing colonoscopy). Additional large multi‐centered studies are needed to fully understand the impact of COVID‐19 on VTE in patients with IBD and to help improve outcomes in such patients.

## Ethics Statement

The National Inpatient Sample (NIS) Database lacks specific patient and hospital identifiers. Hence, an analysis did not require Institutional Review Board (IRB) approval as per the guidelines our institutional IRB provides for analyzing database studies.

## Consent

The National Inpatient Sample (NIS) Database lacks specific patient identifiers. Hence, patient consent was not required.

## Conflicts of Interest

The authors declare no conflicts of interest.

## Supporting information


**Data S1** Supporting Information.
