# Supplementary material for: Impact of COVID‐19 on Venous Thromboembolism in Inflammatory Bowel Disease Hospitalizations: A Propensity‐Matched Analysis
Source: JGH Open. 2025 Jul 11;9(7):e70220. doi: 10.1002/jgh3.70220 (PMC12247105; doi:10.1002/jgh3.70220)
Supplement: Supplementary file 1 — Data S1 Supporting Information. [file JGH3-9-e70220-s001.docx]

ICD 10 CODES:

Coronavirus infection 2019 (COVID-19): J1282, J1281, U071, U00, U49, U50, U85, B342, B9729, B9721

Crohn’s disease: K50

Ulcerative colitis: K51

Sepsis: A40, A41, R652, T8112

Septic shock: R57

Acute respiratory distress syndrome (ARDS): J80

Intubation/mechanical ventilation: 5A1935Z, 5A1945Z, 5A1955Z, 0BH17EZ, 0BH18EZ, 0BH13EZ

Non-invasive ventilation: 5A09357, 5A0935A, 5A09358, 5A09457, 5A09458, 5A0945A, 5A09557, 5A09558, 5A0955A

Vasopressor use: 3E030XZ, 3E033XZ, 3E040XZ, 3E043XZ, 3E050XZ, 3E053XZ, 3E060XZ, 3E063XZ

Use of anticoagulant drugs: Z7901

Atrial fibrillation/flutter: I48

Prosthetic heart valve: Z952, Z953, Z594, 3E043XZ, 3E050XZ, T8281, T82867

History of venous thromboembolism (VTE): Z86718, Z86711, I2782, I82211, I82221, I82291, I825, I827, I82A2, I82B2, I82C2, I82891, I8291

Hypercoagulable states: D685, D686

Left ventricular (LV) thrombus: D685, D686

Coronary artery bypass graft (CABG): 021008, 021009, 02100A, 02100J, 02100K, 02100Z, 021108, 021109, 02110A, 02110J, 02110K, 02110Z, 021208, 021209, 02120A, 02120J, 02120K, 02120Z, 021308, 021309, 02130A, 02130J, 02130K, 02130Z

Percutaneous coronary intervention (PCI): 0270346, 027034Z, 02703D6, 02703DZ, 02703T6, 02703TZ, 02703Z6, 02703ZZ, 027044Z, 0270446, 02704D6, 02704DZ, 02704Z6, 02704ZZ, 0270356, 027035Z, 02703E6, 02703EZ, 0270456, 027045Z, 02704E6, 02704EZ, 0270366, 027036Z, 02703F6, 02703FZ, 0270466, 027046Z, 02704F6, 02704FZ, 0270376, 027037Z, 02703D6, 02703DZ, 027047Z, 0270476, 02704G6, 02704GZ, 0271346, 027134Z, 02713D6, 02713DZ, 02713T6, 02713TZ, 02714ZZ, 02714EZ, 02714FZ, 02714GZ, 02713Z6, 0271356, 0271366, 0271376, 0272346, 02713ZZ, 027135Z, 027136Z, 027137Z, 027234Z, 027144Z, 02713E6, 02713F6, 02713G6, 02723D6, 0271446, 02713EZ, 02713FZ, 02713GZ, 02723DZ, 02714D6, 0271456, 0271466, 027147Z, 02723T6, 02714DZ, 027145Z, 027146Z, 0271476, 02723TZ, 02714Z6, 02714E6, 02714F6, 02714G6, 02723Z6, 02724Z6, 0272456, 027236Z, 02724F6, 02723ZZ, 0272356, 027245Z, 02723F6, 02724FZ, 027244Z, 027235Z, 02724E6, 02723FZ, 0272376, 0272446, 02724ZZ, 02724EZ, 0272466, 027237Z, 02724D6, 02723E6, 0272366, 027246Z, 02723G6, 02724DZ, 02723EZ, 02723GZ, 027247Z, 02734ZZ, 027345Z, 0272476, 02733DZ, 027344Z, 0273356, 02734E6, 02724G6, 02733T6, 02734D6, 027335Z, 02734EZ, 02724GZ, 02733TZ, 0273446, 02733E6, 0273366, 0273346, 02733Z6, 02734DZ, 02733EZ, 027336Z, 027334Z, 02733ZZ, 02734Z6, 0273456, 02733F6, 02733D6, 02733FZ, 027337Z, 02733GZ, 0273476, 027346Z, 02734FZ, 02733G6, 027347Z, 02734G6, 02734F6, 0273376, 02734GZ, 0273466, 4A023NZ, B2151ZZ, B2111ZZ
